# Whole-heart multiparametric optical imaging reveals sex-dependent heterogeneity in cAMP signaling and repolarization kinetics

**DOI:** 10.1126/sciadv.add5799

**Published:** 2023-01-20

**Authors:** Jessica L. Caldwell, I-Ju Lee, Lena Ngo, Lianguo Wang, Sherif Bahriz, Bing Xu, Donald M. Bers, Manuel F. Navedo, Julie Bossuyt, Yang K. Xiang, Crystal M. Ripplinger

**Affiliations:** ^1^ Department of Pharmacology, University of California Davis, Davis, CA, USA.; ^2^ Clinical Pathology Department, Faculty of Medicine, Mansoura University, Mansoura, Egypt.; ^3^ VA Northern California, Mather, CA, USA.

## Abstract

Cyclic adenosine 3′,5′-monophosphate (cAMP) is a key second messenger in cardiomyocytes responsible for transducing autonomic signals into downstream electrophysiological responses. Previous studies have shown intracellular heterogeneity and compartmentalization of cAMP signaling. However, whether cAMP signaling occurs heterogeneously throughout the intact heart and how this drives sex-dependent functional responses are unknown. Here, we developed and validated a novel cardiac-specific fluorescence resonance energy transfer–based cAMP reporter mouse and a combined voltage-cAMP whole-heart imaging system. We showed that in male hearts, cAMP was uniformly activated in response to pharmacological β-adrenergic stimulation. In contrast, female hearts showed that cAMP levels decayed faster in apical versus basal regions, which was associated with nonuniform action potential changes and notable changes in the direction of repolarization. Apical phosphodiesterase (PDE) activity was higher in female versus male hearts, and PDE inhibition prevented repolarization changes in female hearts. Thus, our imaging approach revealed sex-dependent regional breakdown of cAMP and associated electrophysiological differences.

## Teaser

Sex-dependent differences in regional cAMP signaling dictate repolarization changes during sympathetic activity.

## INTRODUCTION

Sympathetic activity is a key contributor to the initiation and maintenance of ventricular arrhythmias (*1*). In particular, heterogeneity of sympathetic activity throughout the heart can be pro-arrhythmic. Anatomical and functional heterogeneity in the form of apico-basal or transmural gradients in innervation, ion channel expression, and action potential duration (APD) can lead to nonuniform sympathetic responses and heterogeneity of repolarization, thereby promoting reentrant arrhythmias (*2*–*5*). The processes that underlie repolarization homogeneity also vary by sex (*6*), and the incidence of and risk factors for a variety of arrhythmias are therefore sex-dependent (*7*). Sex differences in ion channel expression, including fewer K^+^ channels and increased peak L-type Ca^2+^ current in females, result in longer APD and increased vulnerability to early after depolarizations (EADs) (*8*) . In addition, female hearts have a smaller repolarization reserve and a greater dispersion of repolarization, which make females more vulnerable to long QT-related arrhythmias (*9*–*11*). Despite these known sex disparities, there is a lack of mechanistic understanding of the role of biological sex in arrhythmogenesis.

Altered downstream cellular signaling responses to sympathetic activity may also promote arrhythmias (*12*–*15*). Sympathetic responses are finely tuned to rapidly increase cardiac output in response to physiological stress through intracellular cyclic adenosine 3′,5′-monophosphate (cAMP)–dependent protein kinase A (PKA) pathways. cAMP is an important second messenger for sympathetic control, responsible for transducing extracellular autonomic signals to functional inotropic and chronotropic responses. cAMP mediates these responses via PKA phosphorylation of several targets within the cell involved in intracellular Ca^2+^ handling (e.g., ryanodine receptors, phospholamban, and RAD guanosine triphosphatase) and sarcolemmal ion channels (e.g., RAD-dependent regulation of L-type Ca^2+^ channels and K^+^ channels). cAMP activity is tightly regulated by phosphodiesterases (PDEs), which are cAMP-degrading enzymes that play a key role in subcellular cAMP compartmentalization and nanodomain signaling (*12*, *16*, *17*).

The role of cAMP signaling as a key messenger in transducing cellular responses has been widely investigated, primarily with the use of fluorescence resonance energy transfer (FRET)–based reporters in isolated cardiomyocytes. However, the vast majority of studies have focused on subcellular cAMP heterogeneity and compartmentalization (*16*, *18*–*21*), and there is little understanding of macroscale heterogeneity of cAMP signaling throughout the heart, or how cAMP signaling contributes to arrhythmogenesis. Here, we developed a cardiac-specific FRET-based cAMP reporter mouse and a multiparametric approach that enables imaging of FRET-based cAMP activity along with simultaneous optical mapping of transmembrane potential (V_m_) in intact mouse hearts. This powerful approach uses cAMP as a measure of adrenergic cellular signaling responses to sympathetic activity, and membrane potential (V_m_) as a measure of resulting electrophysiological function, to provide the first real-time assessment of how functional electrophysiological outputs respond to cellular signaling events at the whole-heart level. Data revealed faster decay of cAMP in the apex of female mouse hearts following pharmacological β-adrenergic stimulation. We further show that region- and sex-dependent differences in cAMP activity coincide with higher PDE activity in apical regions of female hearts. Moreover, spatial heterogeneity of cAMP activity results in nonuniform APD changes and a marked change of the direction of repolarization in female but not male hearts. Thus, our results indicate regionally heterogeneous breakdown of cAMP as an underlying mechanism contributing to sex-dependent electrophysiological differences, which may play a role in sex-dependent arrhythmia susceptibility.

## RESULTS

### Expression of a cardiac-specific cAMP biosensor in mice

**Fig. 1.**
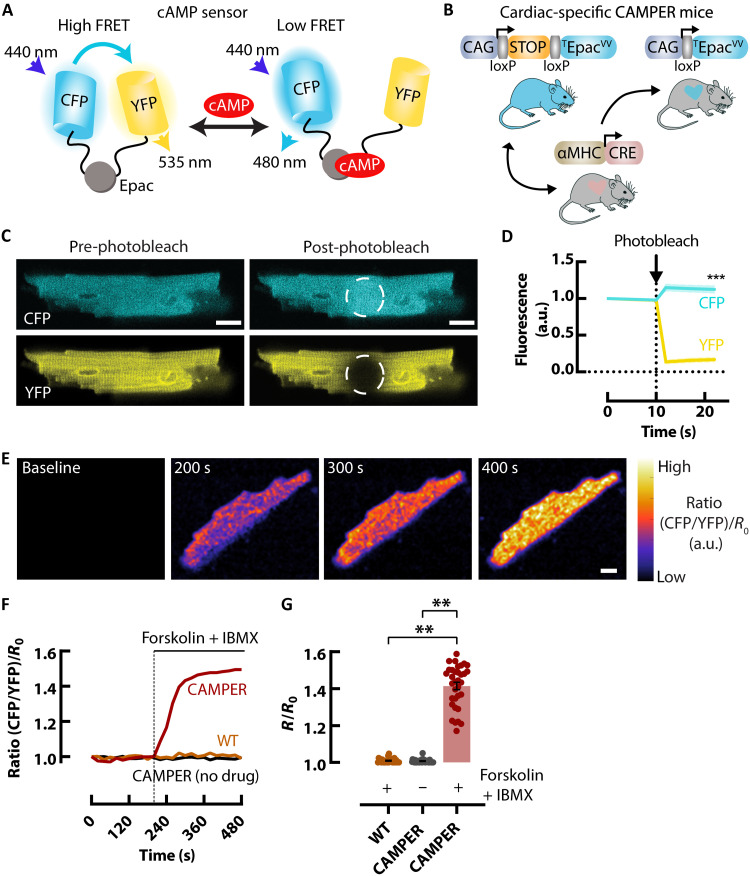
Expression of a cardiac-specific FRET-based cAMP biosensor in mice. (**A**) Schematic diagram of the cAMP FRET biosensor in the cAMP-free and cAMP-bound form. (**B**) Cartoon showing the generation of the cardiac-specific *CAMPER* mouse. (**C**) Isolated ventricular myocytes from the *CAMPER* mouse showing CFP and YFP fluorescence before and after acceptor photobleaching. (**D**) Mean data showing an increase in donor fluorescence following acceptor photobleaching. *N* = 3 hearts (female), *n* = 30 cells total. (**E**) Example pseudo-colored FRET ratio images of *CAMPER* ventricular myocytes before (baseline) and after 25 μM forskolin + 100 μM IBMX. (**F**) Representative FRET ratio traces and (**G**) Mean maximal cAMP response (max ∆FRET) scatter dot plots with (red) and without (black) 25 μM forskolin + 100 μM IBMX in *CAMPER* and wild-type (WT) C57/BL (orange) ventricular myocytes. *N* = 4 *CAMPER* hearts (female), *n* = 35 cells for no drug, *n* = 37 cells for forskolin + IBMX; *N* = 3 wild-type hearts (female), *n* = 26 cells. Individual data points represent cells. Scale bars, 10 μm. ***P < 0.01, by nested one-way analysis of variance (ANOVA) compared to no drug or wild type. a.u., arbitrary units.

To assess sympathetic signaling responses in the intact heart, we used the conditional *CAMPER* reporter mouse (*22*) expressing a FRET-based Epac-mediated cAMP biosensor (Fig. 1, A and B) (*23*). To generate cardiac-specific expression, we crossed the conditional *CAMPER* reporter mouse with an α–myosin heavy chain–Cre mouse (Fig. 1B) (*24*). We first tested the ability of *CAMPER* to report on cAMP activity in isolated ventricular myocytes. Freshly isolated ventricular myocytes from the cardiac-specific *CAMPER* mice exhibited robust cyan fluorescent protein (CFP) and yellow fluorescent protein (YFP) fluorescence (Fig. 1C). After photobleaching of the YFP acceptor, we observed a 15 ± 1.1% increase in CFP donor fluorescence, confirming efficient energy transfer between the two fluorescent proteins (Fig. 1D). To further validate the cardiomyocyte-specific FRET biosensor, the broad adenylyl cyclase activator forskolin and the broad PDE inhibitor 3-isobutyl-1-methylxanthine (IBMX) were added to the cells to maximally activate cAMP (Fig. 1, E to G). Application of forskolin + IBMX in *CAMPER* myocytes increased the CFP/YFP FRET ratio 41.4 ± 1.97%, compared with no drug and forskolin + IBMX in wild-type cardiomyocytes (Fig. 1G). These results confirmed a large dynamic range and the viability of this sensor to assess cAMP activity in the mouse heart.

### Development of a whole-heart multiparametric voltage-FRET imaging system

**Fig. 2.**
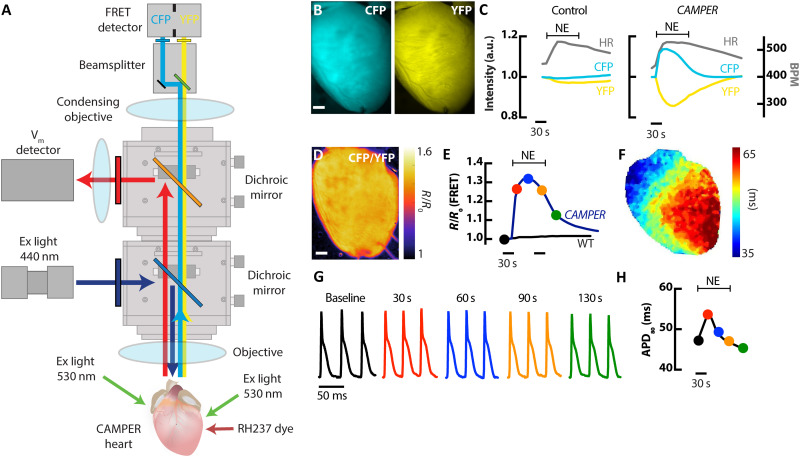
Development of a whole-heart voltage-FRET imaging system. (**A**) Schematic diagram of the macroscale whole-heart FRET and optical mapping system. (**B**) Representative images of a cardiac-specific *CAMPER* mouse heart showing CFP and YFP fluorescence. Scale bar, 1 mm. (**C**) Temporal CFP and YFP fluorescence responses in control and *CAMPER* mouse hearts after application of a 1.5 μM bolus NE with corresponding changes in HR. (**D**) Pseudo-colored CFP/YFP ratio image (scale bar, 1mm) and (**E**) CFP/YFP [normalized to baseline (*R*_0_)] ratio changes in response to 1.5 μM NE. (**F**) Example map of APD (APD_80_) during sinus rhythm. (**G**) Example optical APs from the heart in (D) from the time points marked on the FRET ratio graph in (E) and the APD_80_ graph in (H). (**H**) Temporal APD_80_ changes in response to 1.5 μM NE.

To image cAMP activity in the intact heart of *CAMPER* mice, a THT-macroscope (Scimedia) was fitted with an additional beamsplitter (OptoSplit II Bypass, Cairn) to split CFP/YFP emission onto a single FRET detector (Photometrics Prime) (Figure 2A). CFP was excited at 445 ± 10 nm with a light-emitting diode (LED) epi-illumination light source passing through the sample objective for uniform excitation. For optical mapping of V_m_, a MiCam Ultima-L (SciMedia) detector was used. V_m_ was detected using RH237, which is excited at 531 ± 20 nm with liquid light guides focused directly on the surface of the heart. Because of the partial overlap of RH237 excitation and YFP emission, as well as the vastly different frame rates for V_m_ versus cAMP signals (*F*_s_ = 1.5 ms for V_m_ and 100 ms for cAMP), interleaved imaging was performed. Langendorff-perfused hearts from *CAMPER* mice showed robust CFP and YFP fluorescence (Fig. 2B), which was two to three times higher than autofluorescence of isolated hearts from *CAMPER^+^* Cre^−^ (control) hearts. Application of an acute bolus of norepinephrine (NE; 1.5 μM) in *CAMPER* hearts via the arterial perfusion cannula activated β-adrenergic receptors and led to an increase in CFP and a decrease in YFP fluorescence, resulting in a robust increase in the FRET ratio (Fig. 2, C to E). NE bolus perfusion in control hearts lacking the cAMP reporter had only minor effects on CFP/YFP fluorescence (Fig. 2C), despite robust increases in heart rate (HR) during NE perfusion in both *CAMPER* and control hearts. This important result confirms the FRET signal in the *CAMPER* hearts is much greater than any changes in background autofluorescence that may occur during metabolic challenge (*25*). Action potentials (APs) were optically mapped throughout NE application (Fig. 2F), which showed transient prolongation of APD at 80% repolarization (APD_80_) in response to NE (Fig. 2G), consistent with previous results (*26*). APD_80_ kinetics were plotted over time (Fig. 2H) to assess relationships between cAMP signaling and electrophysiological responses.

### Whole-heart cAMP and AP heterogeneity following β-adrenergic stimulation

**Fig. 3.**
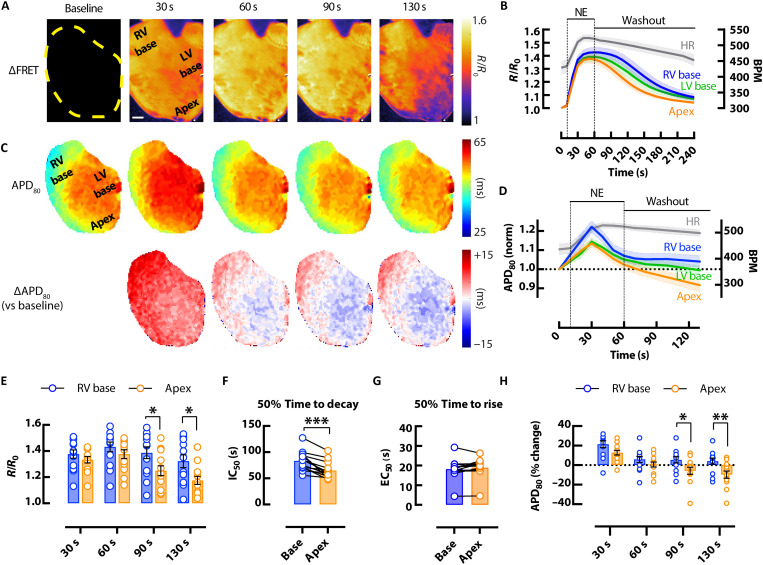
Effects of β-adrenergic stimulation on cAMP responsiveness and APD heterogeneity in the mouse heart. (**A**) Representative CFP/YFP whole-heart ∆FRET ratio images showing the spatiotemporal kinetics of cAMP activity (scale bar, 1 mm) and (**B**) average (mean = solid lines; SEM = shadow) CFP/YFP FRET ratio traces in response to bolus of 1.5 μM NE from different regions of the heart (blue = RV base; green = LV base; orange = apex), with corresponding changes in HR (gray). (**C**) APD_80_ maps from the heart in (A) (top) with change in APD_80_ versus baseline (∆APD_80_) (bottom) and (**D**) average (mean = solid lines; SEM = shadow) normalized APD_80_ over time showing APD prolongation and then shortening after application of 1.5 μM NE, with corresponding changes in HR (gray). Mean scatter dot plots from RV base (blue) and apex (orange) regions following 1.5 μM bolus NE and washout showing; (**E**) FRET ratio responses at different time points; (**F**) FRET ratio 50% decay time calculated from half maximal inhibitory concentration (IC_50_); (**G**) FRET ratio 50% rise time calculated from median effective concentration (EC_50_); and (**H**) APD_80_ % changes from baseline (time 0). *N* = 13 hearts from 6 male and 7 female mice. Representative heart in (A) and (C) from a female mouse. *P < 0.05; **P < 0.01, by two-way ANOVA with multiple pairwise comparisons; ***P < 0.001, by two-tailed paired *t* test.

To assess regional heterogeneity in cAMP signaling and electrophysiological responses, FRET and V_m_ (RH237) imaging during acute bolus NE infusion (1.5 μM) was performed in Langendorff-perfused *CAMPER* mouse hearts of both sexes. Upon NE administration, HR increased 26 ± 4.6% (Fig. 3, B and D). Spatial-temporal kinetics of cAMP (Fig. 3, A and B) and APD_80_ (Fig. 3, C and D) were assessed from three cardiac regions of interest (ROIs) from the right ventricular (RV) base, left ventricular (LV) base, and apex. The greatest heterogeneity in both parameters (cAMP and APD) was observed between RV base and apex regions, with the LV base having intermediate responses. Therefore, the remainder of the analysis focused on comparison between these two regions at different time points during NE infusion and washout. When male and female *CAMPER* hearts were combined, cAMP levels were higher in the RV base compared to the apex, most prominently at 90 and 130 s after bolus application of NE (Fig. 3E) when cAMP levels were declining. No regional differences were observed in the max FRET response during bolus NE infusion at maximal (1.5 μM) or submaximal doses (100 and 500 nM) (fig. S1). Higher levels of cAMP at the RV base during NE washout was accompanied by delayed cAMP decay kinetics in the RV base versus apex at higher doses of NE (Fig. 3F and fig. S1), suggesting that regional heterogeneity exists in the breakdown of cAMP. Despite regional differences in cAMP decay kinetics, there was no regional variability in cAMP rise time (Fig. 3G).

Consistent with previous studies (*26*), we observed a biphasic APD response (prolongation followed by shortening) in all regions of the mouse heart following bolus application of NE. However, significant regional differences in APD_80_ were observed between the base of the heart compared with the apex at 90 and 130 s after bolus application of NE. HR remained elevated (18 ± 4.9%) in all hearts at 130 s. We found that APD_80_ at the base of the heart had a slower return to baseline where cAMP levels remain elevated (Fig. 3H). Thus, the magnitude of changes in cAMP activity at the base versus apex corresponded to functional base versus apex differences in APD_80_. Biphasic APD responses to bolus application of NE were observed in the presence of alpha (α)-1 adrenergic inhibition with 100 nM and 1 μM prazosin (fig. S2), verifying the involvement of a β-adrenergic–driven cAMP-dependent mechanism in APD changes. These findings suggest strong associations between regional changes in ΔAPD and cAMP (FRET) during sympathetic stimulation and withdrawal.

### Sex-dependent differences in cAMP and electrophysiological responses in the mouse heart

**Fig. 4.**
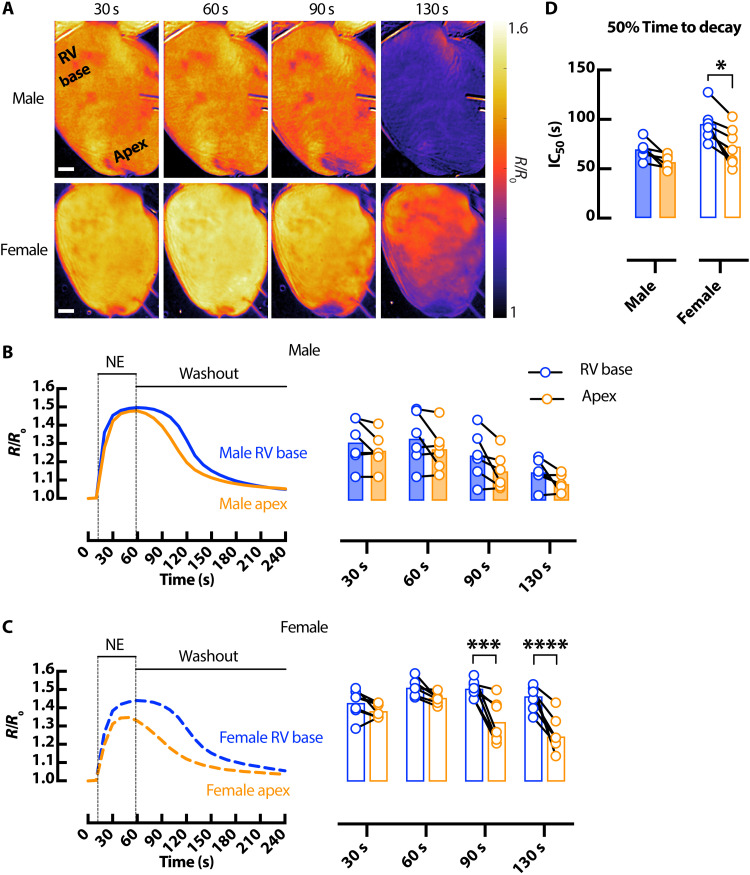
Sex-dependent differences in cAMP spatiotemporal responses in the mouse heart. (**A**) Representative ∆FRET ratio images showing the spatiotemporal kinetics of cAMP activity in male (top) and female (bottom) *CAMPER* hearts after application of 1.5 μM NE. Scale bars, 1 mm. Corresponding representative FRET ratio traces and mean scatter dot plots from RV basal (blue) and apical (orange) regions following 1.5 μM bolus NE at different time points in male (solid) (**B**) and female (dashed) (**C**) *CAMPER* mouse hearts. (**D**) 50% time to decay calculated from FRET ratio IC_50_. *N* = 6 male and 7 female hearts. *P < 0.05; ***P < 0.001; ****P < 0.0001, by two-way ANOVA with multiple pairwise comparisons.

We next asked whether sex is a biological variable in cAMP responsiveness in the intact heart. cAMP signaling was assessed in response to acute bolus infusion of NE (1.5 μM) in male and female *CAMPER* hearts (Fig. 4A). Male hearts demonstrated more uniform rise and fall of cAMP throughout the heart (Fig. 4B), whereas female hearts displayed marked regional heterogeneity in cAMP responses (Fig. 4C). cAMP levels remained elevated in the RV base compared to apex in female hearts at the 90 and 130 s time point following NE application. Furthermore, we observed delayed cAMP decay in the RV base of the heart versus apex in female but not male hearts (Fig. 4D).

**Fig. 5.**
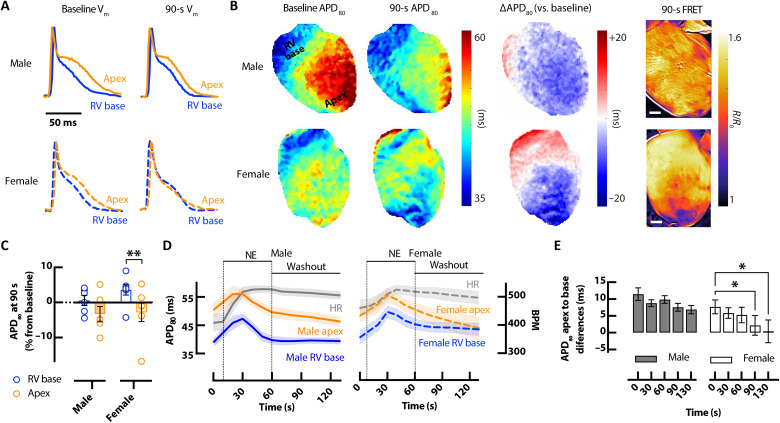
Sex-dependent differences in AP responses. (**A**) Example optical APs at baseline and following bolus of 1.5 μM NE showing shortening of APD in the apex of the female *CAMPER* mouse heart 90 s after NE. (**B**) Example APD_80_ maps (left) with change in APD_80_ at 90 s versus baseline (∆APD_80_) (middle) and corresponding cAMP ∆FRET ratio images at the same time points (right). Scale bars, 1 mm. (**C**) APD_80_% change from baseline (time 0) at 90 s after NE. (**D**) Average (mean = solid lines; SEM = shadow) temporal APD_80_ in response to bolus of 1.5 μM NE from RV base (blue) and apex (orange), with corresponding changes in HR (gray). (**E**) Average APD_80_ apex-to-base differences in male (closed bar) and female (open bar) hearts corresponding to time points in (D). *N* = 6 male and 7 female hearts. *P < 0.05, by two-way ANOVA with multiple pairwise comparisons.

Corresponding sex and regional differences in AP kinetics were also assessed. Before NE infusion, APD_80_ was longer in the apex than RV base in both male and female hearts (Fig. 5, A and B) and increased similarly during the initial phase of NE perfusion (Fig. 5D). Full APD_80_ time series for male and female hearts in response to bolus NE are shown in fig. S3. Unique to the female heart, the apex APD_80_ progressively and substantially declined after NE, to the point that the apico-basal APD gradient was abolished between 90 and 130 s (Fig. 5, A to E). The regional changes in APD_80_ correspond very well with regional cAMP (FRET) levels at the same time point (Fig. 5B). Notably, female apex APD_80_ and cAMP declined more markedly during NE washout, whereas APD_80_ changes in the female RV base and male apex and RV base were more stable and of similar time course throughout NE washout (Figs. 4, B and C, and 5C).

**Fig. 6.**
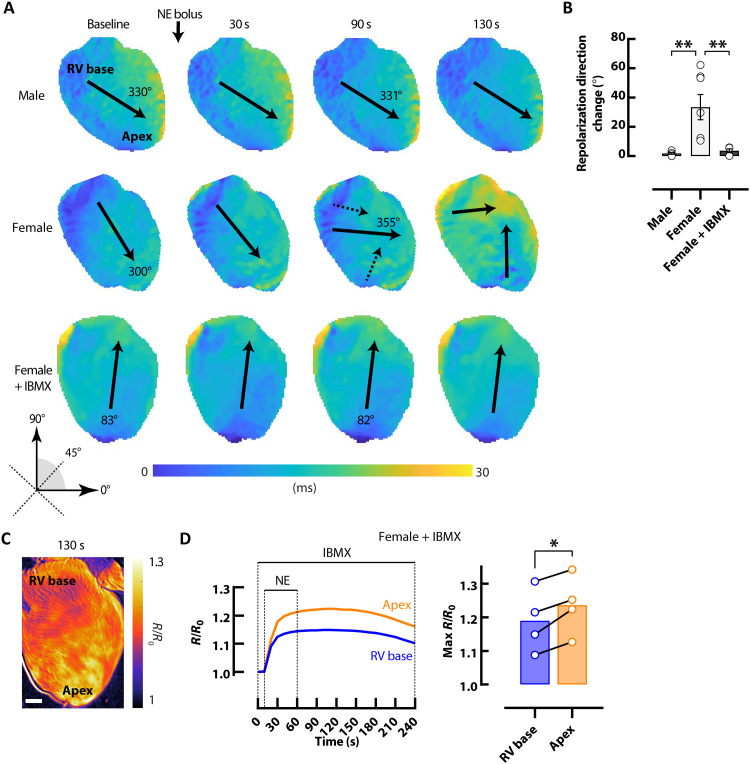
Changes in the direction of repolarization in female hearts. (**A**) Representative repolarization maps demonstrate directional changes in female but not male hearts following bolus of 1.5 μM NE. Repolarization directional changes did not occur with bolus of NE in female hearts following pretreatment with 100 μM IBMX. Solid arrows represent mean repolarization angle. (**B**) Mean angular repolarization direction change with bolus of 1.5 μM NE. *N* = 6 male and 5 to 7 female hearts. (**C**) Representative ∆FRET ratio image showing cAMP activity in a female *CAMPER* heart in response to 1.5 μM NE bolus during perfusion with 100 μM IBMX. Scale bar, 1 mm. (**D**) Corresponding representative FRET ratio traces and mean scatter dot plots from RV basal (blue) and apical (orange) regions following bolus of 1.5 μM NE during perfusion with 100 μM IBMX. *N* = 4 female hearts. *P < 0.05 and **P < 0.01, by one-way ANOVA or paired *t* test.

Regional alterations in APD_80_ kinetics in female hearts in response to acute bolus NE infusion were accompanied by a change in the direction of the repolarization wavefront (Fig. 6, A and B). At 90 s after NE, the mean direction of the repolarization wavefront changed 33.5° ± 1.3° in female hearts, leading to a different repolarization sequence, whereas repolarization direction never changed in male hearts (1.8° ± 0.6°; Fig. 6B). Because the observed sex and regional differences in cAMP signaling and repolarization occurred primarily during cAMP decay, repolarization was assessed during acute bolus NE infusion following pretreatment with IBMX, to inhibit PDEs (Fig. 6, A and B). Following IBMX perfusion, the change in the direction of the repolarization wavefront in female hearts was reduced to 3.7° ± 1.3° during PDE inhibition (Fig. 6B). cAMP signaling responses were also assessed in response to acute bolus infusion of NE during PDE inhibition (Fig. 6, C and D) in female hearts. Maximal cAMP levels during bolus NE infusion were higher in the apex compared to the RV base during PDE inhibition, opposite to regional cAMP responses under control conditions (Fig. 4C).

### Sex-dependent differences in PDE activity in the mouse heart

**Fig. 7.**
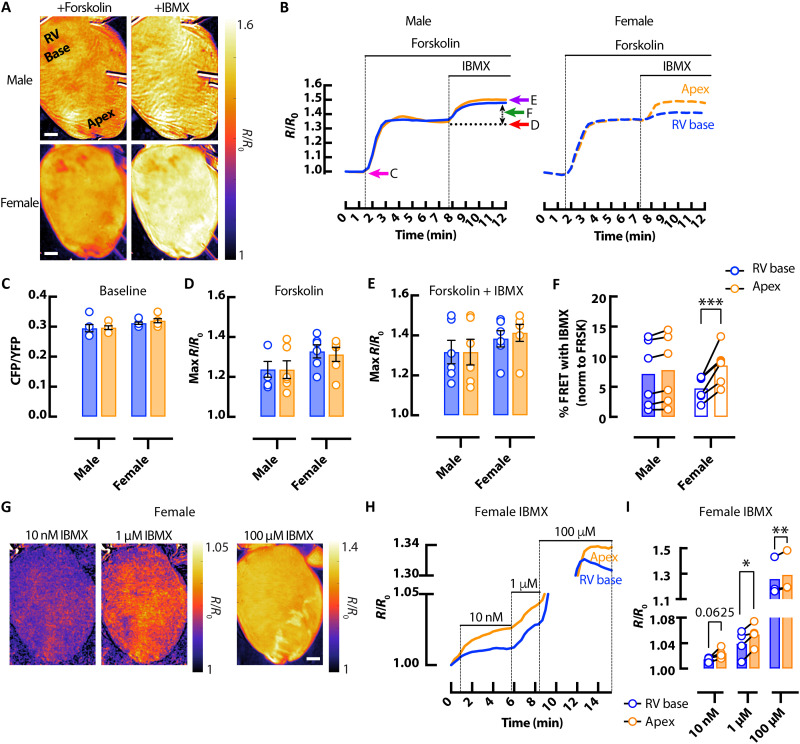
Sex-dependent differences in cAMP breakdown in the mouse heart. (**A**) Representative ∆FRET ratio images showing cAMP activity in male (top) and female (bottom) *CAMPER* hearts after perfusion with 25 μM forskolin then 100 μM IBMX. (**B**) Corresponding representative FRET ratio traces from RV base (blue) and apex (orange) regions in male (solid line) and female (dashed line) *CAMPER* hearts. Mean FRET ratio scatter dot plots showing (**C**) basal cAMP activity (CFP/YFP) before the addition of forskolin + IBMX as indicated by the pink arrow on (B); (**D**) maximal cAMP response to forskolin (max ∆FRET) as indicated by the red arrow on (B); and (**E**) maximal cAMP response to forskolin + IBMX (max ∆FRET) as indicated by the purple arrow on (B). (**F**) cAMP response after the addition of IBMX when normalized to forskolin-stimulated levels, as indicated by the green arrow on (B). *N* = 6 hearts from each group. (**G**) Representative ∆FRET ratio images of cAMP activity after perfusion with submaximal (10 nM and 1 μM) and maximal (100 μM) doses of IBMX. (**H**) Corresponding representative FRET ratio traces and (**I**) mean scatter dot plots from RV basal (blue) and apical (orange) regions following perfusion with submaximal (10 nM and 1 μM) and maximal (100 μM) doses of IBMX. *N* = 4 female hearts *P < 0.05, **P < 0.01, and ***P < 0.001, by two-way ANOVA with multiple pairwise comparisons. Scale bars, 1 mm.

PDE enzymes are responsible for cAMP breakdown. Therefore, we assessed PDE activity in male and female hearts. Baseline cAMP activity (measured by FRET ratio before drug addiction) was not different between male and female hearts (Fig. 7, B and C). Similarly, forskolin-induced cAMP production increased cAMP levels somewhat uniformly across male and female hearts (Fig. 7, A, B, and D). Further increasing cAMP by inhibiting PDEs with IBMX also lead to similar maximal cAMP FRET between sexes (Fig. 7E). However, when the relative change in cAMP activity in response to IBMX was assessed, a greater increase in cAMP activity was observed in apical regions versus the RV base in female but not in male hearts (Fig. 7, A, B, and F). Crucially, regional differences in cAMP responses also occurred at submaximal doses of IBMX (Fig. 7, G to I), without the presence of forskolin in female hearts, suggesting differences in baseline PDE activity throughout the female heart.

**Fig. 8.**
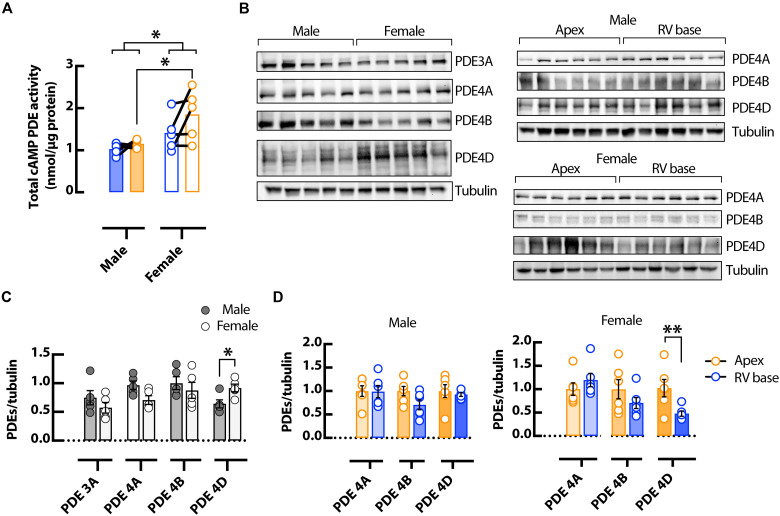
Sex-dependent differences in PDE activity and expression in the mouse heart. (**A**) Total cAMP activity from RV basal (blue) and apical (orange) regions in male and female hearts. *N* = 5 hearts in each group, *P < 0.05, by two-way ANOVA with multiple pairwise comparisons. Representative Western blots (**B**) and normalized mean data (**C** and **D**) for PDE3A, PDE4A, PDE4B, and PDE4D normalized to tubulin expression, in male and female mouse whole ventricles (C) and from RV basal and apical regions (D). *N* = 5 to 6 hearts in each group. *P < 0.05 and **P < 0.01, by one-way ANOVA.

Likewise, total PDE activity assays showed increased apical PDE activity in female versus male hearts (Fig. 8A), suggesting the involvement of PDEs in regionally heterogeneous cAMP signaling in the female hearts. To determine the specific PDE isoforms involved, protein expression of the main murine cardiac PDE isoforms were probed using Western blot (Fig. 8B). No change was observed between male and female hearts in PDE3A, PDE4A, and PDE4B (Fig. 8C), nor between apical and basal regions in PDE4A and PDE4B (Fig. 8D). There was an increase in the expression of PDE4D in female verses male hearts and in female apical verses basal regions (Fig. 8, C and D), suggesting that this isoform may contribute to the sex and regional differences in PDE and cAMP activity observed here.

## DISCUSSION

In this study, we built and validated a novel multiparametric imaging system to assess real-time, whole-heart interactions between cellular adrenergic signaling of local cAMP and the resultant functional electrophysiological outcomes in response to sympathetic activation. To our knowledge, our study is the first of its kind to use such integrated imaging techniques. This approach revealed (i) region-dependent differences in local cAMP activity in female hearts, as shown by faster breakdown of cAMP in the apex of female hearts compared to the base, yet no heterogeneity was found across male hearts; (ii) spatial heterogeneity of cAMP activity in female hearts resulted in nonuniform APD changes and significant changes in the direction of the repolarization wavefront; (iii) female hearts had higher expression of PDE4D compared to male hearts; and (iv) the apex of female hearts had higher total PDE activity and higher expression of PDE4D, which may contribute to faster cAMP breakdown in this region. Together, these data are the first to demonstrate that cellular adrenergic responses to sympathetic activation drive functional electrophysiological outcomes at the tissue level. This is most notable in female hearts, where highly heterogeneous cAMP signaling and PDE activity drive nonuniform changes in APD and repolarization kinetics. These findings are important because arrhythmogenic activity is often attributed to heterogeneous sympathetic responses, and regionally heterogeneous cAMP breakdown via PDEs might be a previously overlooked contributor to sex-dependent differences in arrhythmogenic activity.

cAMP nanodomain signaling and intracellular compartmentalization have been extensively investigated in isolated cardiomyocytes with a variety of FRET-based reporters (*16*, *18*, *20*, *21*, *27*, *28*). However, experiments in isolated cells cannot replicate the spatial heterogeneity of nerve distribution, ion channel expression, and β-adrenergic responsiveness throughout the intact heart. Moreover, experiments in isolated cells cannot predict emergent, tissue-level function such as repolarization gradients or arrhythmogenic activity. To date, two previous studies have used whole-heart cAMP FRET imaging and demonstrated limited regional differences in cAMP responses at the tissue level (*12*, *29*). However, corresponding real-time functional electrophysiological outcomes were not assessed. To address this, we developed a novel multiparametric imaging system capable of imaging cellular cAMP activity along with V_m_ in Langendorff-perfused mouse hearts, where we use cAMP as an intermediate read-out of cellular responses to autonomic activity. Using this approach, we show in the normal female heart, heterogeneous cAMP signaling dictates resulting heterogeneous V_m_ responses. These combined spatiotemporal measurements of cAMP and V_m_ in the intact heart have never been performed and may be key to understanding how pathological autonomic activity is transduced in cardiovascular disease. Notably, our imaging approach is not limited to cAMP and V_m_ and could be applied to virtually any FRET-based biosensor and a combination of V_m_ or intracellular Ca^2+^ or both. Thus, our imaging platform represents a notable advancement in linking cellular signaling activity across spatial and temporal scales to assess resulting functional outcomes in the intact heart.

A key finding of this study was the discovery that degradation of cAMP was nonuniform in female mouse hearts. These findings suggest that cAMP breakdown is modulated differently at different regions of the heart in a sex-dependent manner. Consistent with that, we found increased expression of PDE4D in female verses male hearts, and PDE4D was elevated in the apex versus base of female hearts. Overall PDE activity was also greater in the apex of female hearts. These findings are similar to a previous study showing that baseline cAMP concentration was lower in female verses male cardiomyocytes due elevated levels of PDE4B (*30*). In contrast, Machuki *et al.* (*31*) showed apical myocytes from female mouse hearts had higher baseline cAMP concentration and lower overall PDE mRNA expression verses male. Another study showed no difference in cytosolic cAMP levels between apical and LV basal rat myocytes, although sex differences were not assessed (*12*). These disparities might arise from the experimental models used (rats versus mice), or specificity of regional myocyte isolation, and highlight the regional differences in PDE expression and function and a source of variability when using isolated cell models.

Tissue-level studies have shown, alongside the apico-basal differences in cAMP activity reported here, apico-basal variation in nerve density, β-adrenergic receptor distribution, and ion channel expression (*2*–*5*), indicating that myocytes may be heterogeneously fine-tuned to respond to adrenergic activity to coordinate whole-heart contractile and electrophysiological function. In support of this, an intact heart study from Wright *et al.* (*12*) also showed regional heterogeneity in cAMP and PDE activity. Using a transgenic mouse expressing a plasma membrane–targeted cAMP FRET biosensor (pmEPAC2), the authors detected greater cAMP activation in the apex compared to the LV base following β_2_-adrenergic activation. In contrast to what we report here, higher PDE4 activity was found in LV basal versus apical membrane fractions in this model (*12*). Several factors may underlie these different findings, including that our study primarily assessed β_1_-adrenergic responses (due to the high affinity of NE for β_1_ versus β_2_), we assessed RV rather than LV basal locations (we found LV basal values to be intermediate between apex and RV base; Fig. 3), cellular compartmentalization (plasma membrane versus cytosolic cAMP reporter), and sex differences were not assessed in the study by Wright *et al.* Despite these differences, it is clear that whole-heart region–dependent differences exist in cAMP signaling, which may have substantial functional implications in both normal and diseased hearts. These findings further highlight the importance of novel approaches for determining macroscale heterogeneity of cAMP signaling throughout the intact heart.

During sympathetic activity, APD prolongation can occur if increases in L-type Ca^2+^ current, via PKA phosphorylation of RAD proteins leading to disinhibition of Cav1.2 channels (*17*), outweigh increases in counter-balancing K^+^ currents (*32*), and we have previously shown similar transient APD prolongation in the mouse heart in response to physiological sympathetic nerve stimulation (*26*). Although L-type Ca^2+^ current was not measured in this study, during NE washout, elevated cAMP and prolonged APD in basal regions of female hearts would indicate that L-type Ca^2+^ current remains elevated in this region for a longer period of time following NE application. Previous studies have shown L-type Ca^2+^ current was higher in the base than in the apex of female rabbit hearts, and these differences were not observed in male hearts (*8*). Sims *et al.* (*8*) found that L-type Ca^2+^ current elevation in the base of female hearts promoted EADs in this region. The results of this study therefore suggest that reduced PDE activity and elevated cAMP at the base of the heart in response to sympathetic activity may contribute to sex and regional differences in L-type Ca^2+^ current.

Previous studies have shown marked reversal of the direction of repolarization during physiological sympathetic nerve stimulation in the rabbit heart, but such changes in repolarization did not occur with isoproterenol perfusion (*2*). This is likely due to the spatial gradient of sympathetic innervation (from base to apex), which leads to nonuniform adrenergic stimulation and heterogeneous APD changes during nerve activity, whereas pharmacological adrenergic stimulation is more uniform. A recent study from our group confirmed these findings in rabbit hearts but found that sympathetic nerve stimulation in the mouse heart did not affect the direction of repolarization. Only male animals were assessed in the above studies (*2*, *26*, *33*). In contrast, here, we show that in female mouse hearts, NE perfusion leads to marked repolarization changes, although β-adrenergic receptors are uniformly stimulated throughout the heart with drug application. Repolarization did not change during peak cAMP levels, but rather during NE washout and cAMP decay, suggesting that heterogeneous PDE activity may be a contributor. Supporting this, we demonstrated that when cAMP breakdown was prevented with PDE inhibition, directional changes in repolarization no longer occurred in female hearts. Similarly, in a canine model of Brugada syndrome, inhibition of PDE3 (the major isoform in the canine heart) reduced transmural dispersion of repolarization (*34*). Further investigation is required to determine the effects of PDE inhibition in modulating repolarization kinetics, especially in female hearts.

It remains unknown whether the sex-dependent electrophysiological differences reported here are pro- or anti-arrhythmic. The heterogeneous changes in APD that occurred in female hearts actually tended to reduce the apico-basal APD gradient (Fig. 5E), and decreased APD dispersion can reduce the potential for reentrant arrhythmias (*35*, *36*). Conversely, we observed transient changes in the direction of repolarization in female but not male hearts. Spatiotemporal variability in repolarization is strongly associated with an increased risk for sudden cardiac death (SCD) (*37*). Vectorcardiographic measurements in a large patient population reported increased spatiotemporal repolarization variability in women versus men, and repolarization variability had a stronger association with SCD in women (*37*). Moreover, females in particular are more vulnerable to long QT-related arrhythmias as they have a smaller repolarization reserve and a greater dispersion of repolarization (*9*–*11*). Therefore, it will be essential to investigate the potential role of sex differences in cAMP signaling and PDE activity in models of cardiovascular disease to assess the pro- or anti-arrhythmic effects.

 This study provides real-time assessment of how functional electrophysiological outputs respond to cellular signaling events at the whole-heart level. Using a integrated imaging approach, we determined sex-dependent heterogeneity in cAMP signaling and repolarization kinetics. These results are notable as females are often under-represented in both preclinical and clinical studies. Moving forward, this approach will be useful to assess pathologies such as heart failure, where there are known alterations in cAMP signaling and PDE activity and increased arrhythmias.

### Study limitations

This study used mice, which allowed for in vivo expression of the cAMP biosensor. Mice have known differences in AP kinetics and ion channel expression compared to humans (*38*), and predominant cardiac PDE isoforms may also be different (*39*, *40*). We have previously characterized species differences in AP kinetics in response to sympathetic activity (*26*), and results must be interpreted in this context. It is possible that FRET biosensors may affect myocyte signaling networks (e.g., cAMP buffering via binding to the EPAC sensor), and sensor expression levels may vary between hearts. We primarily assessed changes in FRET signals by normalizing to baseline FRET values (before drug application); therefore, we cannot rule out regional or sex-based differences in adenylyl cyclase activity or baseline cAMP, which were not assessed in this study. Only normal healthy hearts were studied; therefore, direct implications for arrhythmogenesis were not assessed because normal mouse hearts are relatively resistant to ventricular arrhythmias. Therefore, we assessed electrophysiological and repolarization changes and how that might have sex-dependent impacts on arrhythmogenesis; future studies will assess pathological conditions.

## MATERIALS AND METHODS

### Experimental design

To investigate regional adrenergic signaling responses and how these drive electrophysiological outcomes, we built a novel whole-heart multiparametric optical imaging system that enables simultaneous FRET imaging of cAMP signaling events with optical mapping of real-time electrophysiological (V_m_) responses. We have also generated a cardiac-specific reporter mouse that reports cAMP activity via FRET (*CAMPER* mice).

### Ethical approval

All procedures involving animals were approved by the Animal Care and Use Committee of the University of California, Davis (reference no. 22280) and adhered to the Guide for the Care and Use of Laboratory Animals published by the National Institutes of Health (NIH publication no. 85-23, revised 2011). Male (*n* = 10) and female (*n* = 17) *CAMPER* mice (13 weeks old, bred in house at UC Davis) and male (*n* = 17) and female (*n* = 18) wild-type C57BL/6J mice (12 weeks old, The Jackson Laboratory) were house on a 12-hour light-dark cycle and given access to food and water ad libitum.

### Generation of cardiac-specific *CAMPER* mouse

The *CAMPER* floxed mouse (Jackson Laboratories no. 032205), which reports cAMP binding by changes in FRET between a mTurquoise donor and Venus acceptor (due to spectral similarities, spectra are referred to as CFP and YFP, respectively) tagged to the cAMP binding domain of Epac1 (*22*), was crossed with the cardiac-specific α–myosin heavy chain (*Myh6*) Cre mouse (Jackson Laboratories no. 011038) to obtain either *CAMPER*^−/+^ Cre^+/+^ or *CAMPER*^+/+^ Cre^+/+^ breeding pairs, as described previously (*24*). Mice were genotyped before use, and both heterozygous and homozygous male and female *CAMPER* mice 13 weeks old ± 2 days were used for experiments. Fluorescence values from *CAMPER^−/+^* and *CAMPER^+/+^* were not directly compared.

### Ventricular myocyte isolation and imaging

Enzymatic isolation of mouse LV cardiomyocytes was performed as previously described (*41*, *42*). Myocytes were maintained in Tyrode solution containing 1 mM Ca^2+^ and imaged on a fluorescent microscope (Nikon Eclipse Ti, 40× 1.25 numerical aperture or Leica DMI3000 B, 40×). CFP and YFP fluorescence were excited at 445 nm, and emission was measured at 485 ± 15 nm for CFP and 540 ± 15 nm for YFP (Nikon) or at 475 ± 20 nm for CFP and 535 ± 12.5 nm for YFP (Leica). A subset of cells were treated with 25 μM forskolin and 100 μM IBMX (Tocris Bioscience). Enhanced donor fluorescence upon acceptor photobleach using 514-nm laser excitation was used to confirm FRET. Experiments were performed at 23°C. ImageJ software was used for image analysis.

### Whole-heart Langendorff perfusion

Mouse hearts were prepared as described previously (*43*–*45*). Briefly, mice were administered an injection of heparin (100 IU, i.p.) and pentobarbital sodium (>150 mg/kg, i.p.). Hearts were Langendorff-perfused via the aorta with oxygenated (95% O_2_ and 5%CO_2_) modified Tyrode solution [128.2 mM NaCl, 1.3 mM CaCl_2_, 4.7 mM KCl, 1.05 mM MgCl2, 1.19 mM NaH_2_PO_4_, 20 mM NaHCO_3_, and 11.1 mM glucose (pH 7.4)] at 37°C. Hearts were transferred to a heated perfusion chamber where perfusion pressure was maintained at 80 mmHg. Blebbistatin (10 μM; Tocris Bioscience) was added to the perfusate to limit motion artifacts (*46*). Ag/AgCl needle electrodes were positioned in the bath to record an electrocardiogram analogous to a lead I configuration.

### Dual FRET imaging of cAMP and optical mapping of V_m_

*CAMPER* mouse hearts were loaded with the voltage-sensitive dye RH237 (5 μl of 5 mg/ml in dimethyl sulfoxide). The anterior epicardial surface was excited at 445 ± 10 nm for FRET imaging and 531 ± 20 nm for V_m_ imaging using LED light sources (T-LED+, Live Cell Solutions, USA; LEX-2; SciMedia, USA). The emitted fluorescence was collected through a THT-macroscope (SciMedia) and split with a short pass dichroic mirror at 554 nm. The longer wavelength emission, containing the V_m_ signal, was long-pass filtered at >700 nm. The emitted V_m_ fluorescence signals were then recorded using a single complementary metal-oxide semiconductor (CMOS) camera (MiCam Ultima-L; SciMedia) with a sampling rate of 667 Hz and 100 × 100 pixels with a field of view of 10 mm by 10 mm. An OptoSplit II Bypass beamsplitter (Cairn) is used to further split the shorter emission wavelength at 510 nm. Fluorescence is band-pass filtered at 485 ± 10 nm for CFP and at 535 ± 15 nm for YFP and recorded onto a single detector CMOS camera (Photometrics) with a sampling rate of 100 ms and 1024 × 2048 pixels with a field of view of 7.24 mm by 14.5 mm. Hearts were subjected to an acute bolus of NE (100 nM, 500 nM, and 1.5 μM; Sigma-Aldrich) to test responsiveness to β-adrenergic stimulation, followed by perfusion with forskolin (25 μM; Tocris Bioscience) to activate adenylyl cyclase and then IBMX (100 μM; Tocris Bioscience) to inhibit PDEs to determine maximal cAMP activation. Experiments were performed at 37°C under normal sinus rhythm, which allowed for assessments of physiological activation and repolarization patterns.

### Optical mapping and FRET data analysis

For whole-heart FRET data analysis, VisaView (Visitron Sytems GmbH) software was used to acquire CFP and YFP fluorescence every 10 to 20 s for 100 ms. To account for nonuniform baseline fluorescence across the intact heart, as well as the curved imaging surface of the heart, FRET was calculated as *R*/*R*_0_, where *R* = donor/acceptor (CFP/YFP) and *R*_0_ = baseline fluorescence, on a pixel-by-pixel basis and plotted as ΔFRET using MATLAB (MathWorks) software. ImageJ software was used to select ROIs representing RV base, LV base, and apex, and *R*/*R*_0_ was calculated for each region. Average FRET ratio curves and FRET ratio responses at various time points were graphed for each of the ROIs. Optical V_m_ data analysis was performed using ElectroMap software (*47*) as described previously (*45*). Masks of the epicardial surface were selected for whole-heart analysis. A spatial Gaussian filter (3 × 3 pixels) and a baseline drift correction (Top-Hat average) were used to postprocess all fluorescent signals. AP repolarization times were calculated at 80% return to baseline. APD_80_ was calculated as repolarization time minus activation time for 10 consecutive beats and averaged. Following APD calculations, whole-heart APD and repolarization image matrix maps were exported from ElectroMap. Fiducial and anatomical markers were used to align images, and ImageJ software was used to select the same ROIs as the FRET data, representing RV base, LV base, and apex, for APD_80_. FRET ratio data were colocalized with APD values for each ROI to determine relationships between cAMP kinetics and functional outputs. A modified version of Bayly *et al.* (*48*) conduction velocity vector algorithm was used to quantitatively assess the mean angular direction of repolarization.

### PDE activity assays

Male and female mouse hearts (C57BL/6J) were excised and the RV base and LV apex dissected before being snap-frozen in LN2. Protein was extracted in PDE activity assay buffer (Enzo Life Sciences) containing protease (Calbiochem) and phosphatase (Thermo Fisher Scientific) inhibitors. Protein quantity of the homogenates was determined using Pierce BCA Protein Assay Kit (Thermo Fisher Scientific) according to the manufacturer’s instructions. Absorbance of each sample was measured in triplicates at 562 nm (Synergy 2, BioTek), averaged and quantified using the BioTek Gen5 analysis software. Protein homogenates were desalted using Bio-Spin 6 columns (Bio-Rad). Total PDE activity assay was determined using a BIOMOL cyclic nucleotide PDE assay kit (BML-AK800, Enzo Life Sciences) at 37°C. Absorbance of each sample was measured in duplicates at 620 nm, averaged and normalized to total protein concentration.

### Protein detection

Male and female mouse (C57BL/6J) ventricular tissue samples from the RV base and apex were lysed in radioimmunoprecipitation assay buffer with phosphatase and protease inhibitors. Total proteins (50 μg per sample) were separated on SDS–polyacrylamide gel electrophoresis gel and then transferred onto a polyvinylidene difluoride membrane. Membrane was incubated with primary antibody overnight at 4°C and then labeled with IRDye 800CW– or 680RD-conjugated secondary antibodies at room temperature for 1 hour. Images were scanned with ChemiDoc MP Imaging System (Bio-Rad Laboratories, CA), quantified by Image Lab software, and normalized to γ-tubulin. The following primary antibodies were used for immunoblotting: PDE3A (112AP, FabGennix International Inc., Frisco, TX), PDE4A and PDE4B (gifts from M. Conti, University of California at San Francisco), PDE4D (ab14613, Abcam, Cambridge, MA), and γ-tubulin (T6557, Sigma-Aldrich, St. Louis, MO).

### Statistical analysis

Data are presented as means ± SEM from *N* animals. Comparisons were made using Student’s *t* test, paired when effects are within the same heart. For multiple comparisons, analysis of variance (ANOVA) with repeated measures, using Sidak’s post hoc test to adjust for multiple testing errors, was used (GraphPad Prism). Data were considered significant when *P* < 0.05. No data points were omitted from analysis.
