# Supplementary material for: Whole-heart multiparametric optical imaging reveals sex-dependent heterogeneity in cAMP signaling and repolarization kinetics
Source: Sci Adv. 2023 Jan 20;9(3):eadd5799. doi: 10.1126/sciadv.add5799 (PMC9858506; doi:10.1126/sciadv.add5799)
Supplement: Supplementary file 1 — Figs. S1 to S3 [file sciadv.add5799_sm.pdf]

Science Advances
AAAS

Supplementary Materials for

Whole-heart multiparametric optical imaging reveals sex-dependent heterogeneity in cAMP signaling and repolarization kinetics

Jessica L. Caldwell *et al.*

Corresponding author: Crystal M. Ripplinger, cripplinger@ucdavis.edu;
Jessica L. Caldwell, jlcaldwell@ucdavis.edu

Sci. Adv. **9**, eadd5799 (2023)
DOI: 10.1126/sciadv.add5799

This PDF file includes:

Figs. S1 to S3

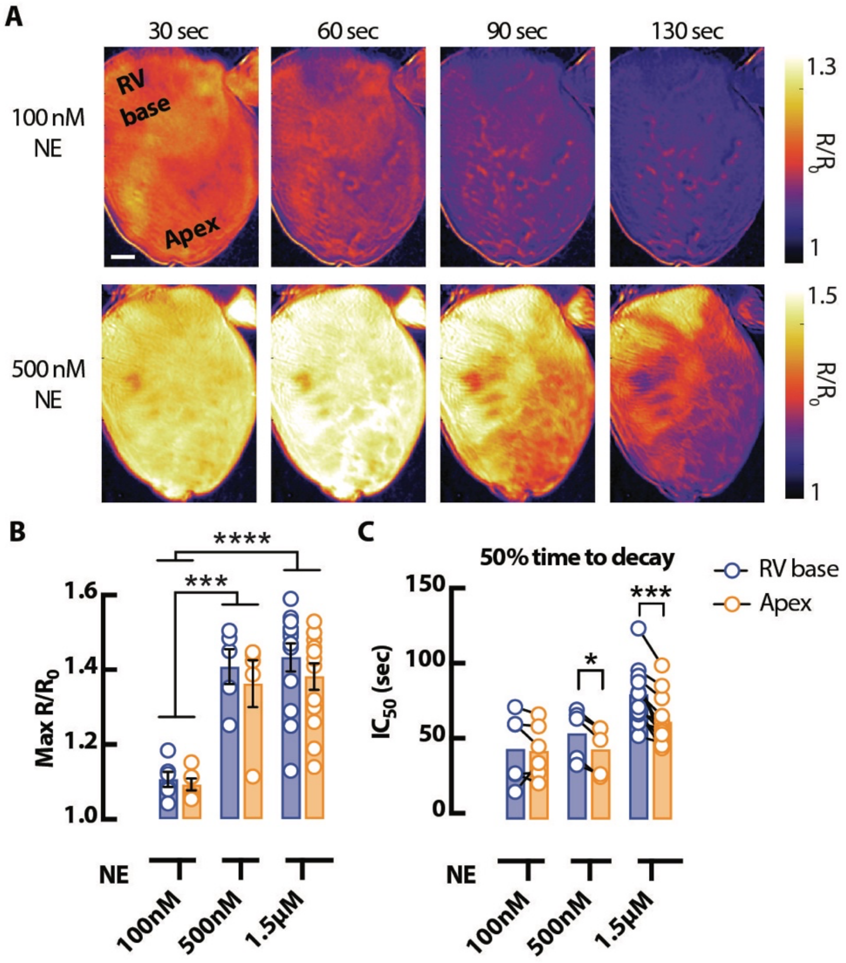

Fig. S1. Effects of submaximal β-adrenergic stimulation on cAMP responsiveness.

(A) Representative CFP/YFP whole-heart Δ FRET ratio images showing the spatio-temporal kinetics of cAMP activity in response to submaximal (100nM and 500nM) bolus of NE. Scale bar = 1 mm. (B) Mean scatter dot plots showing maximal cAMP responses from RV basal (blue) and apical (orange) regions in response to increasing doses of NE bolus (100 nM, 500 nM, and 1.5 μM). (C) FRET ratio 50% decay time calculated from IC_{50} from the same regions in (B). For 100 nM and 500 nM submaximal NE doses, N= 6 hearts from 3 male and 3 female animals. For 1.5 μM, N= 13 hearts from 6 male and 7 female animals. Representative hearts in (A) 100 nM from a male mouse and 500 nM from a female mouse. *$p<0.05$; ***$p<0.001$, ****$p<0.0001$, by two-way ANOVA with multiple pairwise comparisons.

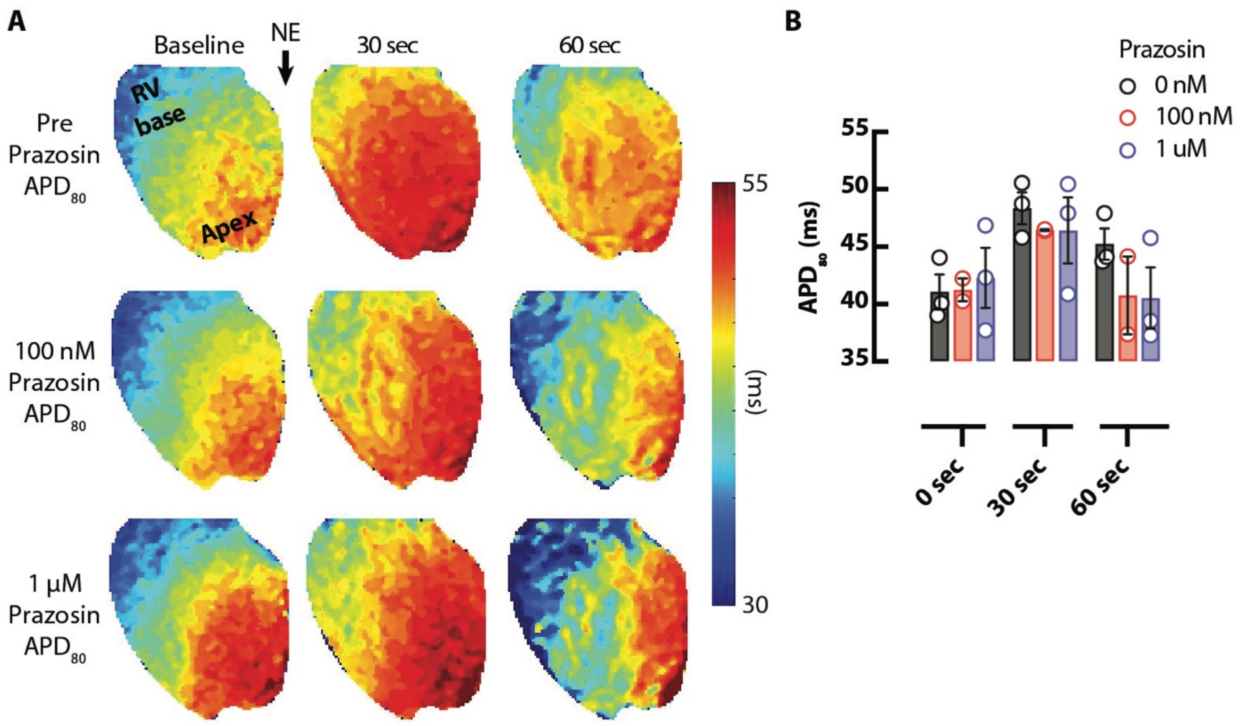

Fig. S2. Effects of β-adrenergic stimulation on action potential duration with alpha-adrenergic receptor inhibition.

(A) Representative APD_{80} maps in response to bolus of 1.5 μM NE before perfusion with the alpha-adrenergic receptor inhibitor prazosin and during perfusion with 100 nM or 1 μM prazosin. **(B)** Mean scatter dot plots showing APD_{80} prolongation at 30 sec post-NE, with or without prazosin and APD_{80} shortening at 60 sec post-NE. APD_{80} measurements were taken from the whole field of view and averaged. For pre- and 1 μM prazosin, N= 3 hearts from 1 male and 2 female animals. For 100 nM prazosin, N= 2 hearts from 1 male and 1 female animal. Representative heart in (A) from a male mouse.

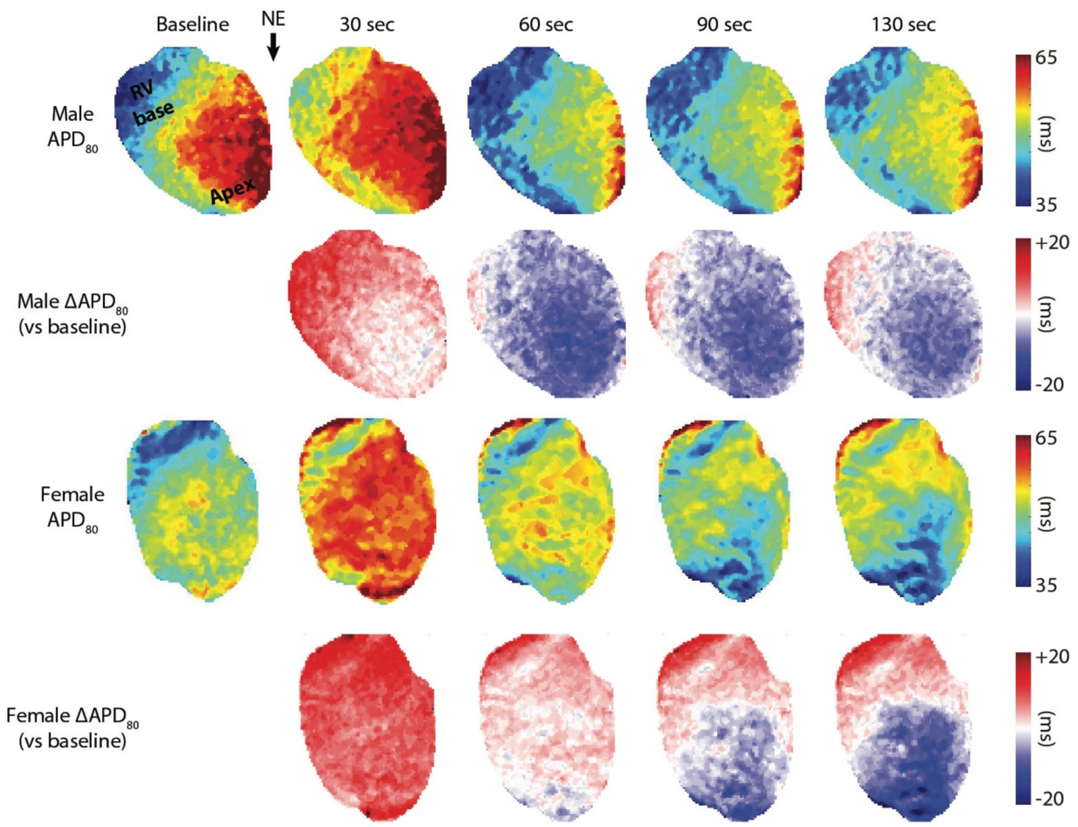

Fig. S3. Representative action potential duration maps in response to β-adrenergic stimulation in male and female hearts.

Representative APD_{80} maps from male and female hearts with change in APD_{80} vs. baseline (Δ APD_{80}) over time showing initial APD prolongation then shortening after bolus application of 1.5 μM NE.
